# Reduction of roadway noise in a coastal city underwater soundscape during COVID-19 confinementa)

**DOI:** 10.1121/10.0003354

**Published:** 2021-01-26

**Authors:** Braulio Leon-Lopez, Eduardo Romero-Vivas, Lorena Viloria-Gomora

**Affiliations:** 1 Acoustics and Signal Processing Research Group, Centro de Investigaciones Biológicas del Noroeste (CIBNOR), Avenida IPN 195, Playa Palo de Santa Rita Sur, C.P. 23096 La Paz, Baja California Sur, Mexico, USA; 2 Programa de Investigación en Mamíferos Marinos, Universidad Autónoma de Baja California Sur (UABCS), Carretera al Sur km 5.5, Mezquitito, C.P. 23080 La Paz, Baja California Sur, Mexico, USA

## Abstract

Confinement due to the COVID-19 pandemic drastically reduced human activities. Underwater soundscape variations are discussed in this study, comparing a typical and confinement day in a coastal lagoon near a popular tourist city in Mexico. Recording devices were located at 2 m in depth and 430 m away from the main promenade—a two-way avenue for light vehicle traffic—where main tourist infrastructure is located. The nearby marine environment is habitat to birds and dolphins as well as fish and invertebrates of commercial importance. Medium and small boats usually transit the area. The main underwater sound level reduction was measured at low frequencies (10–2000 Hz) because of the decrease in roadway noise. Vessel traffic also decreased by almost three quarters, although the level reduction due to this source was less noticeable. As typical day levels in the roadway noise band can potentially mask fish sounds and affect other low frequency noise-sensitive marine taxa, this study suggests that comprehensive noise analysis in coastal marine environments should consider the contribution from nearby land sources.

## I. INTRODUCTION

Human activities are an important noise source in the ocean; thus, assessing their impact on marine fauna is an active research topic,^1^ but efforts have been mainly focused on shipping noise. However, additional to airborne sound that reaches the water, land generated vibrations might be transmitted from structures to the water basement and radiated as sound.^2–4^ The constant growth of coastal urban areas comes with an increase in human activities, which usually involves more marine traffic and also more land traffic. Nevertheless, the actual contribution of roadway noise is not usually addressed. Human water and land activities are constantly happening, and it is rare to document events where they totally stop. The global situation due to the COVID-19 pandemic has led governments to establish confinement rules, which has resulted in the limitation of outdoor activities, night curfews, closure of borders, reduction of flights, and prohibition of non-essential transportation around cities.^5^ Researchers have been taking advantage of this hold on human activities to study the impact on acoustic soundscapes.^6,7^

In Mexico, on 28 February 2020, the first case of COVID-19 was confirmed. An alert system of six levels was set, in which level 6 means strict confinement and level 1 is defined as normality. Strict confinement (level 6/6) started on 30 March and ended on 1 June 2020^8,9^ All education centers and malls and most stores were closed. Only hospitals, pharmacies, and supermarkets were allowed to open with restricted closing times. Additionally, in the municipality of La Paz, Baja California Sur, beaches were closed, and marine navigation was limited to local fishers. Land traffic and commerce activities were completely forbidden along the city coastal avenue with strict vigilance; curfew for car traffic in the rest of the city was set to 22:00 h every day. At the time of writing, level 3 is enforced, and although many commercial activities are allowed, educational centers remain closed, and many people still work from home.^10,11^ The aforementioned situation has provided a unique opportunity to investigate the underwater soundscape of this coastal area during confinement. This baseline can be compared with past days when human activities were typical. Therefore, this study aimed to compare the underwater soundscape for both conditions at a popular tourist destination area in northwestern Mexico and investigate the contribution of roadway noise to the marine environment and its possible implications for marine fauna.

## II. METHODS

### A. Study area and data collection

Ensenada de La Paz is a coastal lagoon connected to La Paz Bay via a 1-km wide natural channel in the Gulf of California. Most of the channel has depths of less than 5 m. However, the central dredged ship canal at the center of the channel goes down to 10 m.^12^ The benthic bottom structure is mainly sandy.^13^

The lagoon takes its name from the capital city La Paz, Baja California Sur, Mexico, which has a coastal 3-km promenade, used for walking and cycling, extending along the southern part of the channel. Alongside, a two-way low speed avenue (60 km/h maximum) is built for light vehicle traffic (vehicles <3855 kg).^14^ This avenue has traffic of around 13 663 cars on a normal day as measured on 3 March 2018.^15^ This downtown area serves as the main harbor drive where most bars, restaurants, and social and cultural activities take place. This popular destination holds international sport fishing tournaments. It has seven private marinas and constant recreational, ecotourism, and fisher traffic, which consist of medium and small vessels (5–30 m length).^16^ During the COVID-19 contingency, authorities have reported a record low in vessel traffic.^16^

Fisher communities in the area depend on commercial species catch, such as white mullet (*Mugil cephalus*), leopard grouper (*Mycteroperca rosacea*), Pacific pen shell (*Atrina maura*), rock bass (*Paralabrax maculatofasciatus*), crab (*Callinectes bellicosus*), sea cucumber (*Isostichopus fuscus*), Pacific calico scallop (*Agropecten ventricosus*), and chocolata clam (*Megapitaria squalida*).^17^ The lagoon is also an important habitat for bottlenose dolphins (*Tursiops truncatus*) that feed and reproduce in the area.^18^ Occasionally, sea lions (*Zalophus californianus*)^19^ and whale sharks (*Rhyncodon typus*) are reported.^20^

Recording analyses from stationary autonomous acoustic systems are shown for a normal day and a day during COVID-19 confinement. A typical day on Wednesday, 13 March 2019, with common land and marine traffic is considered as a normal day. This is the last available recorded day of 2019 in the site. Thanks to fishers in the area, the recorder deployment was made possible once during the COVID-19 pandemic on Friday, 15 May 2020. During that time, land traffic on the coast and ecotourism vessel trips were absent, and only some fishers were allowed to work.

**FIG. 1.**
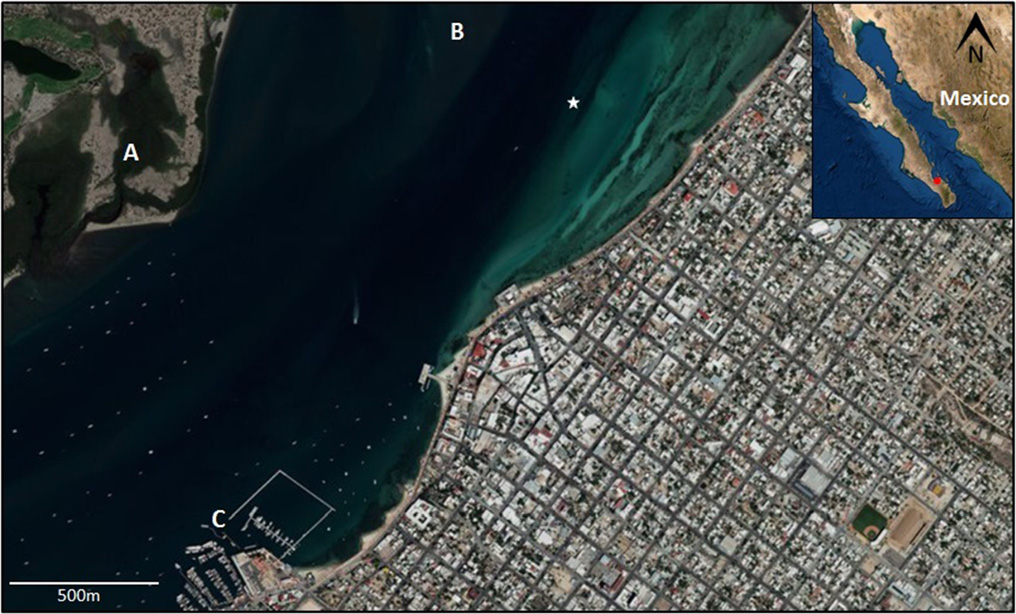
(Color online) Satellite image (ESRI) of the entrance to the Ensenada de La Paz showing the urban area and natural channel: mangroves in the sandbar (A); shallow shoal at the entrance of the channel (B); and marinas (C). The deeper navigation canal can be appreciated as a darker hue in the water. The white star marks the position of the autonomous recorder (24° 10′ 11.3160″ N and 110° 18′ 52.4880″ W) at 430 m from the coast. Boat traffic can be observed in the channel. Inset: Location of La Paz (red dot) in the Baja California peninsula, Mexico.

In both cases, the system is attached to the chain of one of the buoys that signals the navigation channel at 2 m in depth, recording continuously for 24 h starting and ending at 10 a.m. This point, at the entrance of the lagoon at 430 m from the coast (measured from satellite Google Earth®), has been selected from previous studies aiming to monitor bottlenose dolphin presence (Fig. 1). The equipment comprises a sealed plastic recipient containing a SONY ICD-PX470 (Sony Corporation, Tokyo) stereo digital recorder (20–22 000 Hz frequency range, 30 dB gain, 44.1 kHz sampling rate) connecting to an Electrolaer E1 (Electrolaer S.A. de C.V., Mexico City, Mexico) omnidirectional hydrophone (10–100 kHz usable frequency range,^21^ −205 ± 3 dB re 1 V/μPa sensitivity).

Additionally, after the strict confinement was lifted, but still on a level 3/6 alert, a motor vehicle traffic count was performed on the coastal avenue in front of the recording site. One sample hour in the morning (08:00–09:00 h) and another one at night (22:00–23:00 h) for four days (Monday–Thursday) were used as proxy for determining “typical” car traffic rate and ground truth of noise source.

**FIG. 2.**
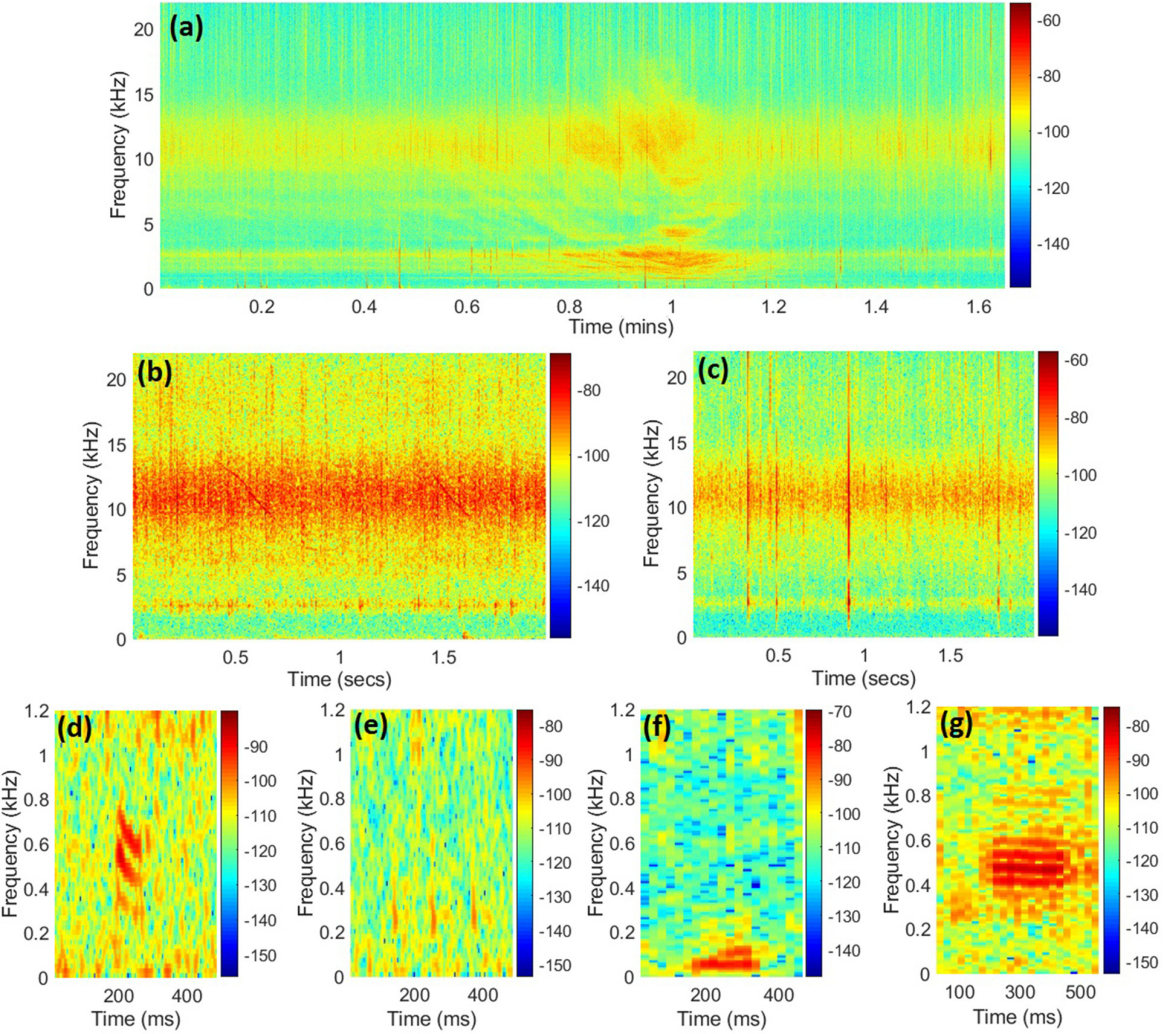
(Color online) examples of anthropogenic and biological sounds detected in the recordings: (a) vessel noise; (b) dolphin whistle; (c) snapper shrimp pulses; (d)–(g) fish sounds. Vertical axes indicate frequency (kHz), horizontal axis indicates time, and the color bar indicates power/frequency (dB/Hz). Spectrograms were created using a Hann window, 50% overlaps. Window size differs for better quality image: (a) 4096; (b) and (c) 512; (d) and (e) 1024; (f) and (g) 2048.

Sound sources were detected visually and aurally in spectrograms using audacity 2.3.2 (GNU GPL). Vessels were identified by Lloyd's mirror effect and tonal engine components [Fig. 2(a)], searching on a 22 050 Hz bandwidth, Hann window size 4096, 50% overlap. The duration of each vessel detected was estimated from Lloyd's mirror interference pattern considering the 2× data window period (DWP) before and after the closest point of approach (CPA) according to ANSI-ASA_S12.64_PART_1 (vessel noise standards).^21^

Dolphin whistles were identified as tonal modulated signals^22^ [Fig. 2(b)]. Whistles were searched on a 22 050 Hz bandwidth, Hann window size 512, and 50% overlap. Fish sounds were identified based on their qualitative characteristics on the spectrogram, considering the most common types attributed to soniferous fish, whether pulsed, wideband, or harmonic within the most common fish production bandwidth (<1 kHz)^23,24^ [Figs. 2(d)–2(g)]. Fish sounds were searched on a 1500 Hz bandwidth, Hann window size 2048, and 50% overlap. Snapping shrimp pulses [Fig. 2(c)] were not counted as they occur constantly throughout the recordings and are considered as part of the typical tropical estuary soundscape.

### B. Data analysis

Calibrated power spectrum density (PSD) profiles and long-term averaged spectrograms (LTASs) were computed using pamguide^25^ in matlab 2013 (Mathworks Inc.) (1-s Hann window, 50% overlap). Those analyses were performed for each 24 h (typical and confinement day) and at high traffic hours in the morning (08:00–09:00) and low traffic at night (22:00–23:00) for both conditions. Statistical noise levels (L*n*), representing the noise level exceeded *n*% of the time, were computed at 1%, 10%, 90%, and 99%. L1 and L99 were chosen to highlight the highest and lowest levels reached, L10 as indication of the upper limit of fluctuating noise. This measured inland value for roadway noise correlates well with the disturbance felt by people next to roads and was included for future comparison with land measurements in which this value is usually reported. Equally, L90 was used as an indicator of background noise.^26^

## III. RESULTS

**FIG. 3.**
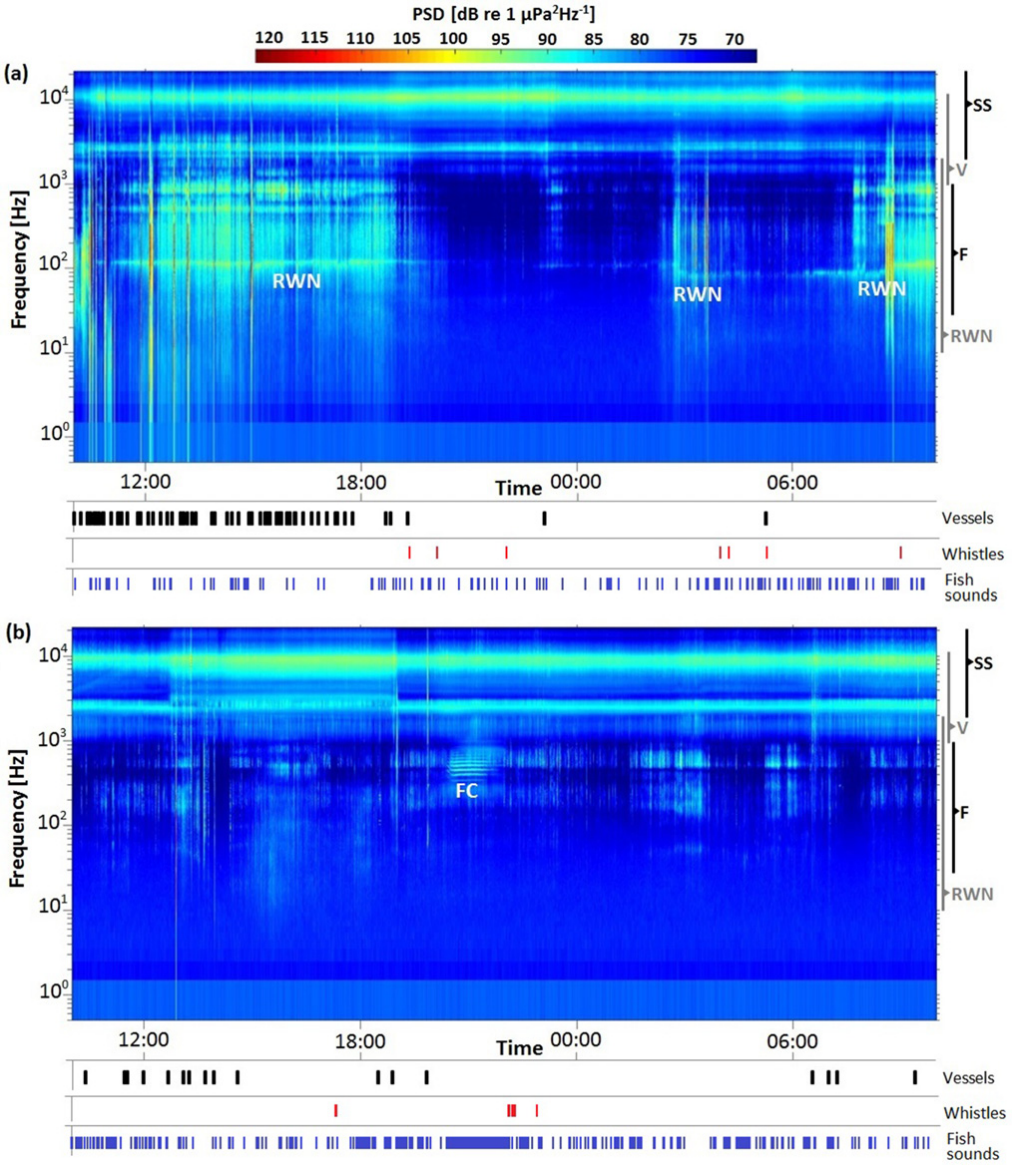
(Color online) LTASs of both conditions showing 24 h from 10:00 h. (a) Typical day, 13 March 2019. (b) Pandemic confinement, 15 May 2020. On the bottom of each spectrogram, vessels, dolphin whistles, and fish sound detections are shown as vertical short black, red, and blue lines, respectively, in time. Frequency bands of some sources are also shown on the right side: snapping shrimp frequency band (SS); fish sound frequency band (F); detected vessel noise band (V); and roadway noise frequency band (RWN). Roadway noise and a fish chorus (FC) are marked on the spectrogram.

The LTAS of a typical day shows a strong noise contribution at the low frequency band around 10–1000 Hz [Fig. 3(a)]. On the other hand, a lack of high energy noise bands at low frequency was observed during the confinement with the exception of putative fish sounds, such as a clear chorus [Fig. 3(b)].

The number of vessels detected and time with vessel noise were 7.18 times lower during confinement than during a typical day, with almost 4 times fewer vessels navigating (Table I).

**TABLE I.** Number of vessels detected and time with vessel noise in each of the conditions.

Condition	Number of vessels	Time with vessel noise (min)	Average vessel duration (min)
Typical day	61	81.65	1.41
Confinement	17	11.37	0.71

Dolphin whistle detections were just slightly different in both samplings (Table I), with a few more whistles detected during the confinement day. On the contrary, fish sound detections and time with fish sound were much higher than during the confinement day, mostly due to the long fish chorus (Table II) detected in the LTAS [Fig. 3(b)] at around 21:00 h. During the typical day sample, no long choruses were detected. Other than in a continuous chorus, fish sounds tend to be too short in time to be clearly noticeable on the LTAS.

**TABLE II.** Number of dolphin whistles and fish sounds detected in each of the conditions.

Condition	Number of dolphin whistles	Time with dolphin whistles (s)	Number of fish sounds	Time with fish sounds (s)
Typical day	17	16.79	203	22.09
Confinement	29	24.68	4737	2288.16

Higher frequency bands around 2.5 and 10 kHz are dominated by snapping shrimp noise, present at all times during both days.

**FIG. 4.**
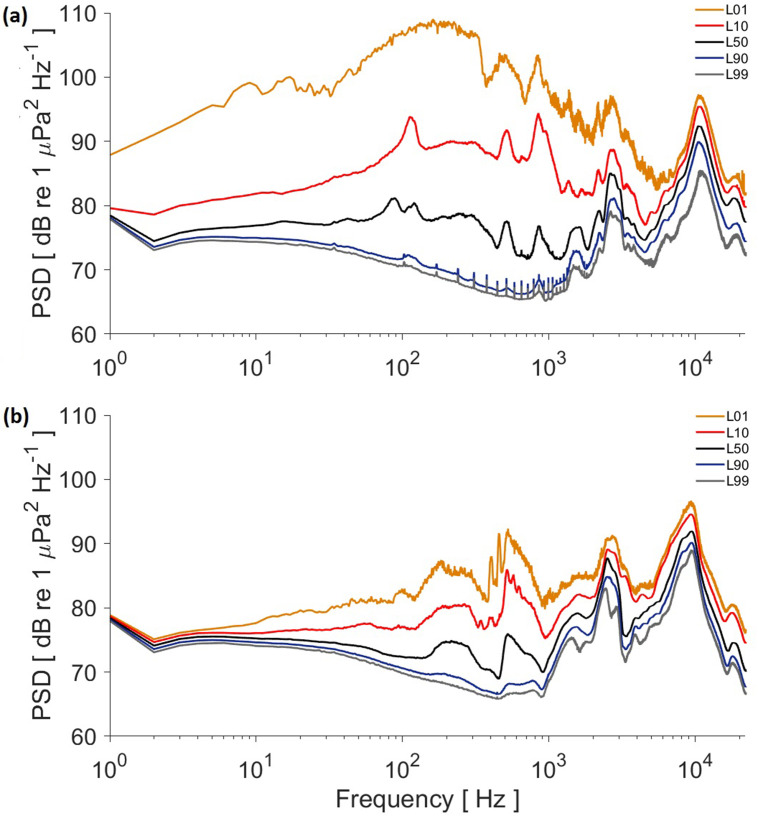
(Color online) Power spectrum density analysis for each condition recording: (a) Typical day and (b) pandemic confinement day. Each line is an equivalent level: L01, L10, L50, L90, and L99.

The difference among 24 h statistical noise levels L01 and L10 within a typical day reflected the presence of intermittent sources [Fig. 4(a)]. In this case, band 10–2000 Hz shows the highest levels. For example, at 200 Hz, L01 and L10 exceed L50 by 30 and 12 dB, respectively. Moreover, small peaks in band 100–2000 Hz can be appreciated at L90 and L99, indicating an almost continuous source of noise at that band. Vessel noise contribution can be observed in the roughness and level difference between L01 and L10 against L50 in the 1–10 kHz frequency band. In the upper band, snapping shrimp dominate, and its natural but lower variability is observed in all levels around its peak at 10 kHz. However, at its lowest peak at 2.5 kHz, snapping shrimp and vessel noise overlap, so a higher variability is observed. On confinement day, those intermittent sources have a drastic reduction in band 10–2000 Hz and a complete absence of the peak on L90 and L99 in the 100–2000 Hz band, showing the lack of the almost continuous noise source found on the typical day [Fig. 4(b)].

The average vehicles per hour, as proxy of a typical day, was 757.5 in the morning, while it was 289.75 at night (Table III), a reduction of 62% in traffic.

**TABLE III.** Number of vehicles in 1 h, morning and night sampling after strict confinement.

Time	Monday	Tuesday	Wednesday	Thursday	Average
08:00–09:00 h	676	754	812	788	**757.50**
22:00–23:00 h	285	274	308	292	**289.75**

**FIG. 5.**
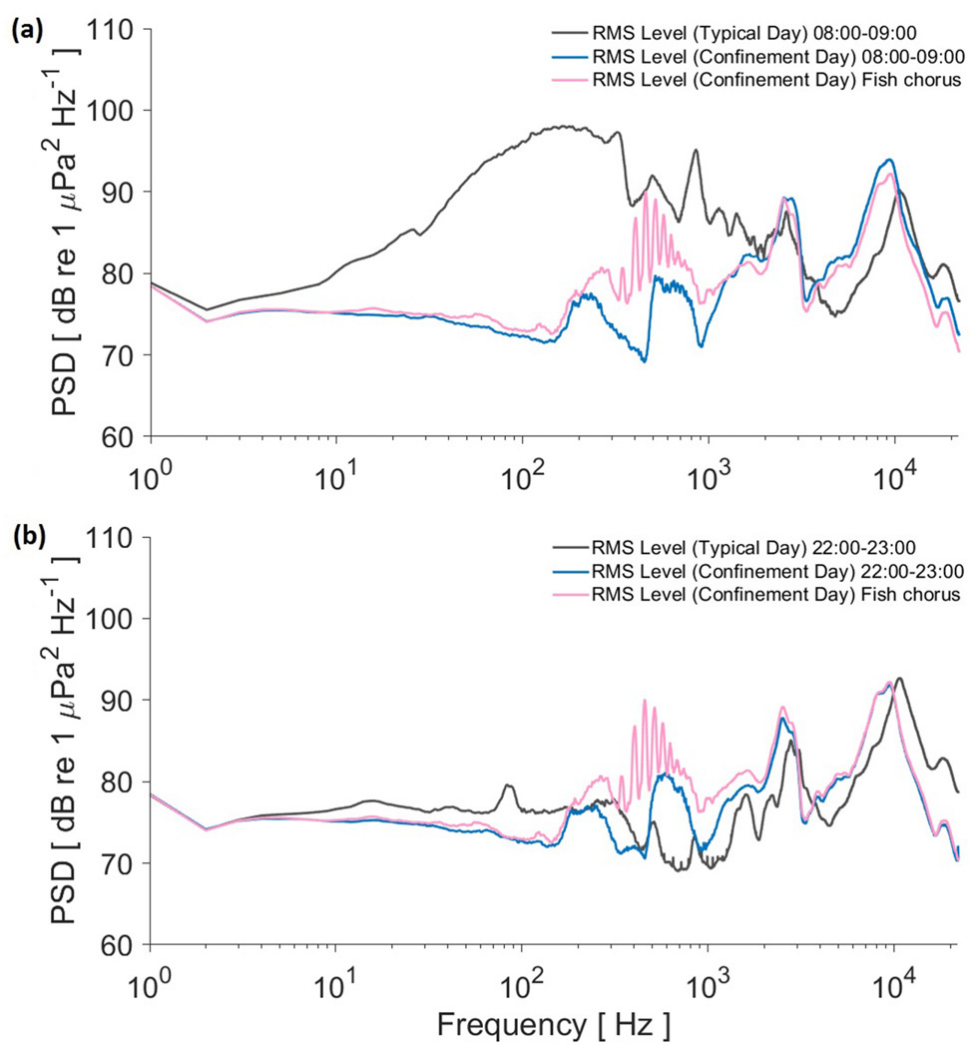
(Color online) Comparison of the rms values from the PSD analyses: (a) 8:00–9:00 h, gray line for the typical day, blue line for the pandemic confinement, and pink line for the fish chorus during confinement day; (b) 22:00–23:00 h, gray line for the typical day, blue line for the pandemic confinement, and pink line for the fish chorus during confinement day.

Comparison of root mean square (rms) values from the PSD level for morning and night hours in both conditions shows that the noise at the 10–2000 Hz band during the pandemic confinement is overall lower (Fig. 5), which is consistent with the drastic human activity reduction and land traffic prohibition.

The difference at band 10–2000 Hz can be clearly noticed in the rms spectra for the typical day between morning and night. For example, rms value at 200 Hz goes from 97 dB in the morning to 76 dB at night, a difference of 21 dB. On the other hand, during confinement day at 200 Hz, the values for morning (76.3 dB) and night (76.2 dB) are almost identical, as they are most of the whole spectra. At the same frequency, a difference of 21 dB is also observed when comparing morning hours between typical and confinement days [Fig. 5(a)]. For night hours, both days are around 76 dB for 200 Hz, but it is even lower for the confinement night in the 10–100 Hz band at 100 Hz, which is 72 dB, while for a typical day, it is 76 dB [Fig. 5(b)].

The example fish chorus was composed of harmonic short sounds [Fig. 2(g)]. Most power for those fish sounds covers the band between 300 and 800 Hz, as shown in the spectrum used for comparing both conditions. The chorus values can be seen above the power levels in the morning and at night of the confinement day but not in the morning of the typical day.

**FIG. 6.**
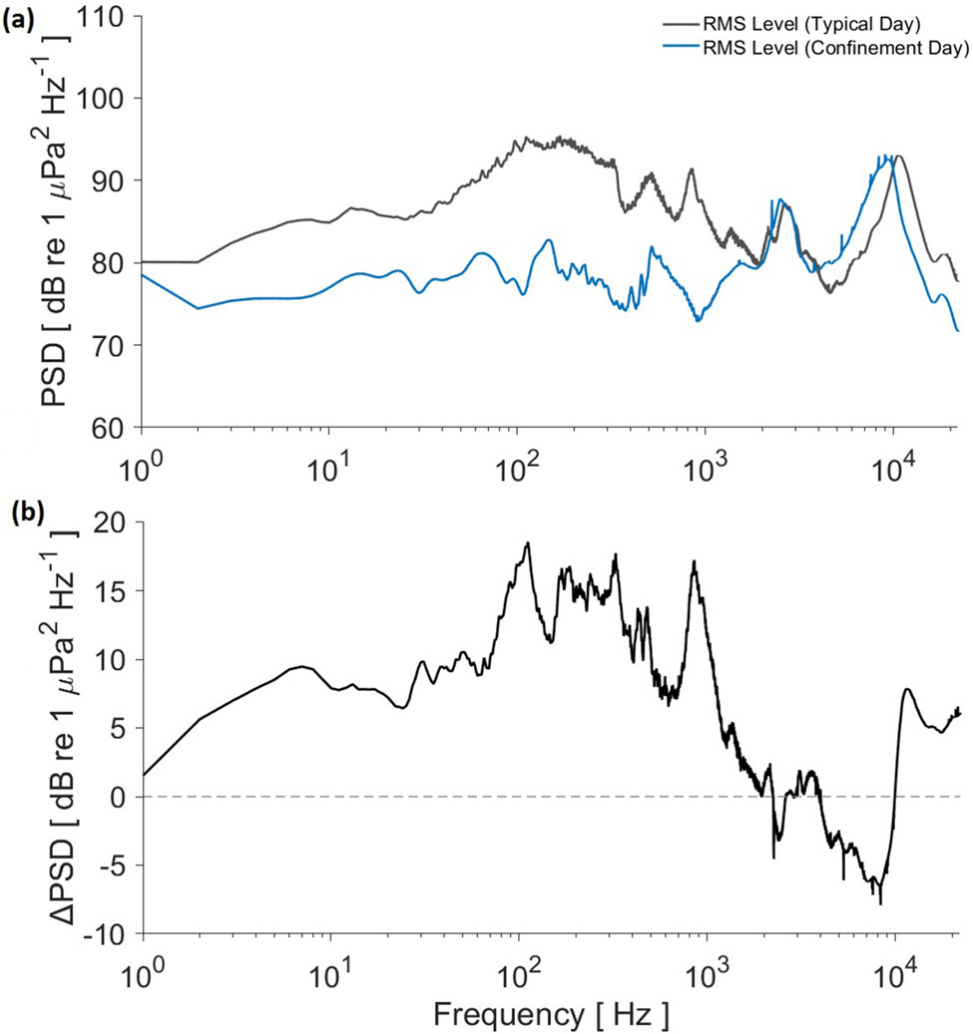
(Color online) Comparison of the rms values from the 24 h PSD analyses. (a) Gray line for the typical day, blue line for the pandemic confinement. (b) Difference (typical day values minus confinement day values) of the rms values from the 24 h PSD analyses.

The comparison of the rms values for the whole 24-h recording shows a general reduction in the contribution to the underwater soundscape during the confinement [Fig. 6(a)]. This difference is clearly shown in frequencies below 2 kHz when subtracting the confinement values from the typical day spectrum, with only a band between 3 and 10 kHz showing more power during confinement than a typical day. However, that switches again once the frequency is over 10 kHz. An even higher difference, of over 10 dB, can be noticed on the band from 50 to 1000 Hz [Fig. 6(b)] with peaks up to 18.5 dB.

## IV. DISCUSSION

The results show a clear difference in the underwater soundscapes during both conditions, especially at low frequencies. In the LTAS analysis, the low frequency noise found during the typical day [Fig. 3(a)] corresponds with car traffic, coinciding with typical working hours, going from the morning up to sunset, when most land traffic slows down as people go back home. This contribution is most likely from roadway noise, both structure-borne and airborne noise (band 10–2000 Hz).^27^ From midnight to early morning, no clear roadway noise is recorded, with the exception of some noise at around 03:00 h, when many bars close and people drive back home. Regular traffic starts again around 07:00 h [Fig. 3(a)]. On the contrary, during confinement days, the car traffic frequency band is notably quieter across the 24 h [Fig. 3(b)], which agrees with total closure of the coastal harbor drive and promenade with the exception of a few police trucks monitoring the area. The PSD levels below 5 kHz show a clear reduction during the pandemic confinement (Fig. 4) because of the reduction of car and vessel traffic; these levels are shown in L01 with a maximum difference at 200 Hz that goes from 108 dB in a normal day to 85 dB during confinement and L10 from 90 dB to 80 dB. Below 2 kHz, during COVID-19 confinement, L01 and L10 show natural sources that may have been masked by roadway noise during a normal day. Furthermore, the peaks on the 100–2000 Hz band for L90 and L99 are showing roadway noise continuous contribution [Fig. 4(a)], which represents how that noise source can be found continuously by a large portion of the typical day even if very high levels only occur intermittently, as shown in its corresponding LTAS [Fig. 3(a)].

A strong reduction of vessels was recorded in the area during confinement (Table I). Nevertheless, vessel noise contribution to the soundscape is less noticeable in the rms PSD because vessels were present less than 6% of a normal day and less than 1% for COVID-19 confinement. This result does not mean that vessel noise lacks the potential of affecting marine organisms as it has been shown in other studies,^28,29^ but it may occur at specific moments or encounters rather than as a large contribution to the general underwater soundscape. Nonetheless, vessels are still detectable in the roughness of L01 in band 1–5 kHz [Fig. 4(a)]. The number of detected vessels was drastically reduced (71% less) during confinement, since only fishers were allowed to work (Table II). This reduction in vessel noise contribution is reflected in the underwater soundscape in that frequency band.

Fish sound detections were fewer than during the confinement day (Table II). Land-borne and waterborne anthropogenic sounds overlapping the fish sound band may potentially be masking detections or even affecting fish distribution along the coast. The fish sound production and hearing band overlaps with the roadway noise band, as most fish species hear best between 30 Hz and 1 kHz, with some being able to detect higher frequencies.^30,31^ A potential masking effect by roadway noise on fish sounds is shown when comparing the rms PSD spectra of the fish chorus section during the confinement day with the rms spectra in the morning and at night during the typical day [Fig. 5(a)]. The morning hours show a value over the whole fish chorus spectrum, potentially masking the whole band, which seems less problematic at night, as the levels for the fish chorus are partially above the expected roadway noise. During the confinement day [Fig. 5(b)], neither the values during the morning nor at night are high enough to potentially mask that example chorus. This similarity is expected as the complete stop of land and marine traffic may erase the difference between morning and night during confinement.

The chorus seems to be produced likely by members of the family Batrachoididae, probably from the genus *Porichthys* sp. which inhabits La Paz Bay.^32^ Species of this family produce harmonic sounds for courtship,^33^ similar to those found in the chorus [Fig. 2(e)]. Therefore, those calls are important for their overall reproductive fitness. Fish in general use sounds in various contexts,^23^ and even though hearing and production frequency bands vary from species to species, those bands clearly overlap with roadway noise.

Snapping shrimp peaks are more consistent in all levels, since no noticeable roadway noise and little vessel noise contribution are recorded at that band. The band above 2 kHz, PSD levels for both morning and night rms spectra have similar levels, although a shift from the 10 kHz peak to 8 kHz is observed during the confinement day and also noticeable in the PSD analysis for the whole 24 h [Fig. 6(a)]. This shift in the snapping shrimp noise band is the main reason for the increase in power values in 3–10 kHz band and sudden decrease after 10 kHz for the confinement day [Fig. 6(b)], even when a large section the band corresponding to roadway noise shows a decrease in over 10 dB for the confinement day. Variations in snapping shrimp sound are more related to natural phenomena, such as tides or temperature,^34^ than to human activity. The typical day analyzed was recorded in late winter, while the recording for confinement happened in late spring, so temperature differences may have been a factor for the variation, rather than the absence of marine and land traffic. Dolphin whistle detections have a small difference between the typical and confinement days. Nevertheless, the most common production band (3–20 kHz)^22^ lies above roadway noise contribution, which seems unlikely to be masking their communication range, although a negative effect on fish may lead to an indirect effect on dolphins and fisher communities that exploit the area for food.

## V. CONCLUSION

Few opportunities exist to study a soundscape without its anthropogenic noise contribution. During the COVID-19 pandemic, human activities have been brought to a halt, providing a unique opportunity to document basal environmental conditions. Vessel noise contribution is usually regarded as the main source of noise for marine environments affecting aquatic life.^35^ However, in water bodies near coastal cities, land traffic may be an important source of noise. During confinement, roadway noise decreases more than 10 dB in the band between 100 and 800 Hz from typical levels considering the whole day—a more noticeable reduction than vessel noise for this locality. Moreover, when comparing roadway noise exclusively in the morning time, that difference is even higher (21 dB at 200 Hz). Continuous low frequency irregular noise has been claimed to increase stress levels and induce communication masking for fish.^36^ Therefore, roadway noise that overlaps with the fish hearing and sound production band is a potential stressor. Low frequency noise may affect invertebrate larval development and behavior as well,^37,38^ but its extent remains still unknown because of the lack of audition curves.^4^ Additionally, a lack of understanding about particle motion detection in fish and invertebrates exists. Even though this study focuses only on sound pressure level, it is important to highlight the necessity of standardized particle motion measurements alongside to assess the effect of land noise contribution to underwater habitats.^39,40^ Studies on the effects of anthropogenic noise in underwater environments are usually focused on vessel noise and marine mammals.^4^ However, in studies related to fish and invertebrates, low frequency roadway noise may be a more important source in shallow marine environments near coastal cities.
